# Supplementary material for: Hybrid Models and Biological Model Reduction with PyDSTool
Source: PLoS Comput Biol. 2012 Aug 9;8(8):e1002628. doi: 10.1371/journal.pcbi.1002628 (PMC3415397; doi:10.1371/journal.pcbi.1002628)
Supplement: Text S4 — Complete source code for the PyDSTool package (version 0.88.120504). Includes API documentation and help files linking to web pages. This file is identical to the current public release on Sourceforge.net. (ZIP) [file pcbi.1002628.s004.zip › PyDSTool/html/PyDSTool.PyCont.Continuation.HopfCurveOne-class.html]

xml version="1.0" encoding="ascii"?


PyDSTool.PyCont.Continuation.HopfCurveOne


| Home | Trees | Indices | Help | PyDSTool |
| --- | --- | --- | --- | --- |

Package PyDSTool ::
Package PyCont ::
Module Continuation ::
Class HopfCurveOne

[hide private]

[frames] | no frames]

# Class HopfCurveOne

source code

```
  object --+    
           |    
Continuation --+
               |
              HopfCurveOne
```

---

```
Child of Continuation class that represents curves of Hopf
points.

Augmented system h(x,a): Uses double bordering on the matrix A
given by

        A = 2*f_{x}(x,a) (*) I

    where (*) denotes the bi-alternate matrix product.

    (see class PyCont.TestFunc.BiAltMethod)
    (see class PyCont.TestFunc.BorderMethod)
    (see class PyCont.TestFunc.Hopf_Double_Bor_One)

    The continuation variables are (x,a) with

        x = State variables
        a = Free parameters

    and the continuation curve is defined by

        h(x,a) = { f(x,a) = 0
                 { det(G) = 0

Detection of points: BT, ZH, GH, DH
```


|  |  |
| --- | --- |
| Instance Methods | [hide private] |

|  |  |
| --- | --- |
| \_\_init\_\_(self, model, gen, automod, plot, \_args=None)  x.\_\_init\_\_(...) initializes x; see x.\_\_class\_\_.\_\_doc\_\_ for signature | source code |

|  |  |
| --- | --- |
| update(self, args)  Update parameters for HopfCurveOne. | source code |

|  |  |
| --- | --- |
| \_createTestFuncs(self)  Creates processors and test functions for HopfCurveOne class. | source code |

|  |  |
| --- | --- |
| \_bialttoeig(self, X, V) | source code |

**Inherited from `Continuation`**:
`__copy__`,
`__deepcopy__`,
`__repr__`,
`__str__`,
`backward`,
`cleanLabels`,
`computeEigen`,
`display`,
`exportGeomview`,
`forward`,
`getSpecialPoint`,
`info`,
`reset`,
`testdomain`,
`testdomaingrid`,
`testdomaintangrid`

**Inherited from `Continuation`** (private):
`_Keller`,
`_MoorePenrose`,
`_Natural`,
`_checkForBifPoints`,
`_compute`,
`_curveToPointset`,
`_preTestFunc`,
`_savePointInfo`,
`_system`,
`_systemjac`,
`_systemjac_withpars`,
`_systemjacuser`,
`_systemuser`

**Inherited from `object`**:
`__delattr__`,
`__getattribute__`,
`__hash__`,
`__new__`,
`__reduce__`,
`__reduce_ex__`,
`__setattr__`


|  |  |
| --- | --- |
| Properties | [hide private] |

**Inherited from `object`**:
`__class__`


|  |  |
| --- | --- |
| Method Details | [hide private] |

|  |  |
| --- | --- |
| \_\_init\_\_(self, model, gen, automod, plot, \_args=None)  *(Constructor)* | source code |

x.\_\_init\_\_(...) initializes x; see x.\_\_class\_\_.\_\_doc\_\_ for
signature

Overrides: object.\_\_init\_\_: *(inherited documentation)*

|  |  |
| --- | --- |
| update(self, args) | source code |

Update parameters for HopfCurveOne.

Overrides: Continuation.update

|  |  |
| --- | --- |
| \_createTestFuncs(self) | source code |

Creates processors and test functions for HopfCurveOne class.

Note: In the following list, processors are in PyCont.Bifpoint and
test functions are in PyCont.TestFunc.

Point type (Processor): Test function(s)
----------------------------------------------

BT (BTPoint): BT\_Hopf\_One DH (DHPoint): DH\_Hopf ZH (ZHPoint): Fold\_Det
(or Fold\_Bor) GH (GHPoint): GH\_Hopf\_One

Overrides: Continuation.\_createTestFuncs

  


| Home | Trees | Indices | Help | PyDSTool |
| --- | --- | --- | --- | --- |

|  |  |
| --- | --- |
| Generated by Epydoc 3.0.1 on Fri May 4 15:24:07 2012 | http://epydoc.sourceforge.net |
